# Supplementary figures and images for: Therapeutic effects of favipiravir against severe fever with thrombocytopenia syndrome virus infection in a lethal mouse model: Dose-efficacy studies upon oral administration
Source: PLoS One. 2018 Oct 26;13(10):e0206416. doi: 10.1371/journal.pone.0206416 (PMC6203377; doi:10.1371/journal.pone.0206416)

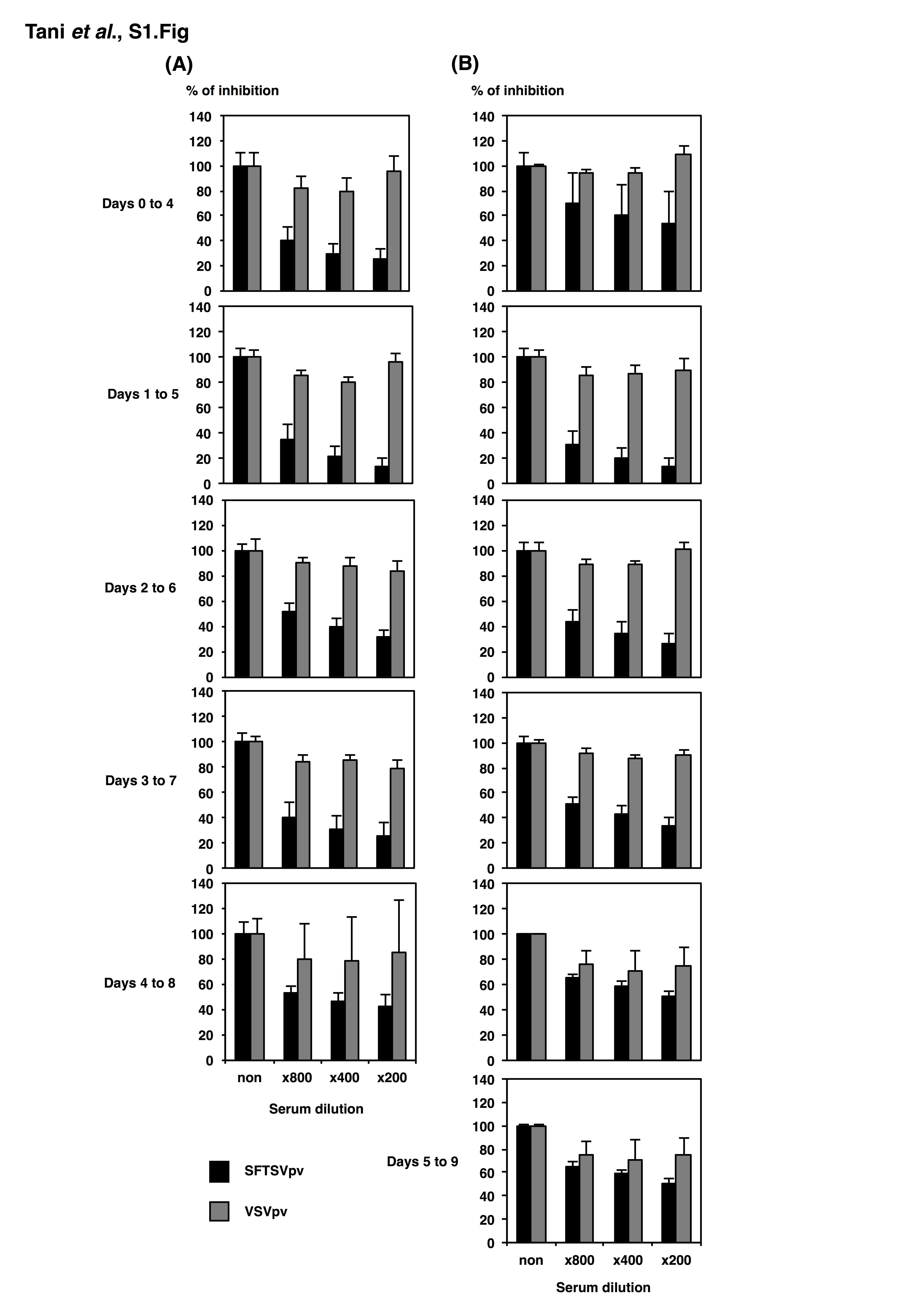

Supplement: S1 Fig — SFTSVpv were preincubated with 200-, 400-, and 800-fold diluted mouse sera collected on Day 14 (120 mg/kg/day treatment group [(A) left columns] and 200 mg/kg/day treatment group [(B) right columns]). Subsequently, Vero cells were infected with SFTSVpv. Infectivity of SFTSVpv was determined by measuring luciferase activities at 24 h post infection. Results from three independent assays are shown, with error bars representing standard deviations. Significance was determined in comparison to the results from non-serum treatment or infectivity of VSVpv. (TIFF) [file pone.0206416.s001.tiff]
